# Advanced Scene Perception for Augmented Reality

**DOI:** 10.3390/jimaging8100287

**Published:** 2022-10-17

**Authors:** Jason Rambach, Didier Stricker

**Affiliations:** Department of Augmented Vision, German Research Center for Artificial Intelligence, Trippstadter Str. 122, 67663 Kaiserslautern, Germany

Augmented reality (AR), combining virtual elements with the real world, has demonstrated impressive results in a variety of application fields and gained significant research attention in recent years due to its limitless potential. AR applications heavily rely on the ability to accurately understand the user’s surroundings as well as dynamically monitor the user’s interactions with their environment. While traditional AR relies on the precise localization of the user, nowadays, a deeper scene perception at multiple levels is expected, ranging from dense environment reconstruction and semantic understanding to hand–object interaction and action recognition. An advanced, efficient understanding of the surroundings is found in AR applications that enable a complete interaction between real and virtual elements and are able to monitor and reliably support users in real-world complex tasks, such as industrial maintenance or medical procedures.

In this Special Issue, we aim to feature novel studies that advance the state-of-the-art research on scene perception for AR applications, contributing to topics such as semantic SLAM, object pose estimation and tracking, dynamic scene analysis, 3D environmental sensing and sensor fusion, hand tracking and hand–object interaction, illumination and reconstruction. Comprehensive state-of-the-art reviews on relevant topics and innovative AR applications that take advantage of recent scene perception developments are also welcome.

The Special Issue includes a total of seven accepted peer-reviewed articles of great significance to the scientific community. Among these articles, four feature novel research results, while three present comprehensive surveys and reviews of AR-related topics. Key topics include self-localization and object pose estimation for AR, as well as user-experience-related issues, such as interaction and specific application areas.

Among these novel research papers, Gupta et al. [1] stress the need to move from 2D object detection towards 3D object detection for AR application purposes. Their approach suggests starting with 2D detection and generating 3D cuboid proposals for the detected objects. Their implementation builds upon the ARCore framework and is designed to be used on mobile devices.

Outahar et al. [2] proposed a combination of direct and indirect visual simultaneous localization and mapping (vSLAM) methods for achieving improved tracking quality on AR applications. Their research is motivated by the fact that direct and indirect methods each show superior performance on entirely different types of scenes. They used an indirect SLAM system as their base and a direct SLAM system mainly for initialization and re-localization. Their experimental results on several benchmark datasets show that the fusion method can be more accurate compared to traditional methods.

Madeira et al. [3] describe the concept of pervasive AR as an extension of traditional AR to experiences that are continuous in space, aware of and responsive to the user’s context and pose. They introduce a process of acquiring 3D scans of an environment that are then used in an AR-experience editing environment. Finally, they investigate the differences between a desktop experience with a 3D model and a fully immersive mobile AR experience in terms of user evaluation. Although the AR interface was generally considered more intuitive, the desktop platform showed promise in several aspects, such as remote configuration, lower required effort, and overall better scalability.

Firintepe et al. [4] are concerned with the highly interesting application of AR within constrained environments, such as a car interior. They suggest that the pose of the AR glasses can be accurately estimated using an IR camera with an outside-in tracking approach. Their approach generates a point cloud of the person wearing the glasses from the IR image and uses this to precisely estimate the pose.

Three highly valuable survey papers were accepted in this Special Issue. Gorschlüter et al. [5] present a survey on 6DoF object detection and pose estimation, which has become a key topic of AR and robotic applications in recent years. They provide an industrial application perspective and focus their review on the methods that exclusively use synthetic data from 3D models of the object for training. A collection of experimental results from different sources regarding the accuracy and runtime performance of these models provides a comprehensive image of the state-of-the-art applications in the field.

In another survey paper, Marto et al. [6] provide a systematic survey on AR games and presence. The survey was conducted following the PRISMA methodology, carefully analyzing all studies that reported visual games that include both AR activities and, somehow, presence data or related dimensions that may be referred to as immersion-related feelings, analysis or results. They describe that immersion-related feelings reported in AR games, in addition to presence, are social presence, co-presence, social immersion, engagement, and self-engagement.

Finally, Nikolaidis [7] provides a meta-review of AR by surveying the topics that have been most surveyed in AR since 2010. A taxonomy of the results is introduced, and the findings mainly reveal the lack of AR application reviews covering all suggested criteria. The results show that existing AR reviews mainly address the areas of healthcare and education, with a much smaller percentage covering industrial topics. The main purpose of this article is to discover the unexplored areas of AR in order to motivate future research by the AR research community.

## References

[1] Gupta N., Khan N.M. (2022). Efficient and Scalable Object Localization in 3D on Mobile Device. J. Imaging.

[2] Outahar M., Moreau G., Normand J.-M. (2021). Direct and Indirect vSLAM Fusion for Augmented Reality. J. Imaging.

[3] Madeira T., Marques B., Neves P., Dias P., Santos B.S. (2022). Comparing Desktop vs. Mobile Interaction for the Creation of Pervasive Augmented Reality Experiences. J. Imaging.

[4] Firintepe A., Vey C., Asteriadis S., Pagani A., Stricker D. (2021). From IR Images to Point Clouds to Pose: Point Cloud-Based AR Glasses Pose Estimation. J. Imaging.

[5] Gorschlüter F., Rojtberg P., Pöllabauer T. (2022). A Survey of 6D Object Detection Based on 3D Models for Industrial Applications. J. Imaging.

[6] Marto A., Gonçalves A. (2022). Augmented Reality Games and Presence: A Systematic Review. J. Imaging.

[7] Nikolaidis A. (2022). What Is Significant in Modern Augmented Reality: A Systematic Analysis of Existing Reviews. J. Imaging.

